# Integrating AI-powered text mining from PubTator into the manual curation workflow at the Comparative Toxicogenomics Database

**DOI:** 10.1093/database/baaf013

**Published:** 2025-02-20

**Authors:** Thomas C Wiegers, Allan Peter Davis, Jolene Wiegers, Daniela Sciaky, Fern Barkalow, Brent Wyatt, Melissa Strong, Roy McMorran, Sakib Abrar, Carolyn J Mattingly

**Affiliations:** Department of Biological Sciences, North Carolina State University, Toxicology Building, 850 Main Campus Drive, Raleigh, NC 27695, USA; Department of Biological Sciences, North Carolina State University, Toxicology Building, 850 Main Campus Drive, Raleigh, NC 27695, USA; Department of Biological Sciences, North Carolina State University, Toxicology Building, 850 Main Campus Drive, Raleigh, NC 27695, USA; Department of Biological Sciences, North Carolina State University, Toxicology Building, 850 Main Campus Drive, Raleigh, NC 27695, USA; Department of Biological Sciences, North Carolina State University, Toxicology Building, 850 Main Campus Drive, Raleigh, NC 27695, USA; Department of Biological Sciences, North Carolina State University, Toxicology Building, 850 Main Campus Drive, Raleigh, NC 27695, USA; Department of Biological Sciences, North Carolina State University, Toxicology Building, 850 Main Campus Drive, Raleigh, NC 27695, USA; Department of Biological Sciences, North Carolina State University, Toxicology Building, 850 Main Campus Drive, Raleigh, NC 27695, USA; Department of Biological Sciences, North Carolina State University, Toxicology Building, 850 Main Campus Drive, Raleigh, NC 27695, USA; Department of Biological Sciences, North Carolina State University, Toxicology Building, 850 Main Campus Drive, Raleigh, NC 27695, USA; Center for Human Health and the Environment, North Carolina State University, Toxicology Building, 850 Main Campus Drive, Raleigh, NC 27695, USA

## Abstract

The Comparative Toxicogenomics Database (CTD) is a manually curated knowledge- and discovery-base that seeks to advance understanding about the relationship between environmental exposures and human health. CTD’s manual curation process extracts from the biomedical literature molecular relationships between chemicals/drugs, genes/proteins, phenotypes, diseases, anatomical terms, and species. These relationships are organized in a highly systematic way in order to make them not only informative but also scientifically computational, enabling inferential hypotheses to be formed to address gaps in understanding. Integral to CTD’s functionality is the use of structured, hierarchical ontologies and controlled vocabularies to describe these molecular relationships. Normalizing text (i.e. translating raw text from the literature into these controlled vocabularies) can be a time-consuming process for biocurators. To facilitate the normalization process and improve the efficiency with which our scientists curate the literature, CTD evaluated and integrated into the curation process PubTator 3.0, a state-of-the-art, AI-powered resource which extracts and normalizes from the literature many of the key biomedical concepts CTD curates. Here, we describe CTD’s long-standing history with Natural Language Processing (NLP), how this history helped form our objectives for NLP integration, the evaluation of PubTator against our objectives, and the integration of PubTator into CTD’s curation workflow.

**Database URL**: https://ctdbase.org

## Introduction

Since 2003, the Comparative Toxicogenomics Database (CTD; https://ctdbase.org) has been a publicly available resource that seeks to address knowledge gaps with respect to how environmental exposure to chemicals affects human health [1].

**Figure 1.**
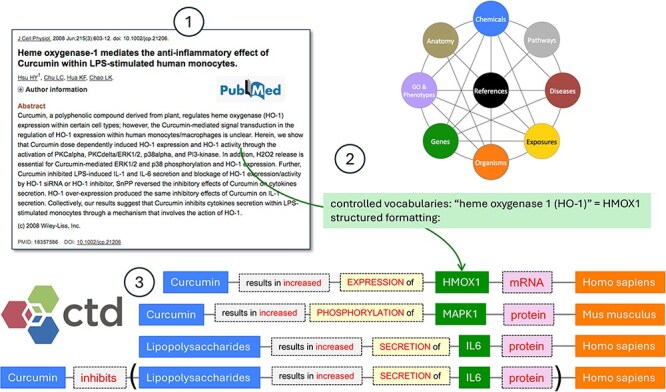
CTD Curation Overview. CTD biocurators review the literature (circle 1), transforming authors’ free text using a specific methodology to make it informative and computational. Well-defined controlled vocabularies are used for every biological aspect of CTD, including chemicals, genes, phenotypes, diseases, anatomical terms, pathways, exposures, and species, as well as edges which represent the relationship between terms. Here, the CTD biocurator normalizes the free text *heme oxygenase 1* and *HO-1* to the NCBI Gene primary symbol *HMOX1* (circle 2). Curation is rendered in CTD using a highly structured, systematic methodology (circle 3).

The foundation upon which CTD is built is the manual curation of peer-reviewed biomedical literature by PhD-level biocurators; we structure free-form text in very specific ways to make it informative and computational. CTD’s core curation module is composed of highly specialized edge relationships between chemical/drugs (referred to hereafter simply as chemicals) and gene/proteins (referred to hereafter simply as genes); chemicals and phenotypes; genes and diseases; and chemicals and diseases. These carefully constructed relationships are defined based on detailed biocurator review of the literature (Fig. 1); each relationship is associated with a specific PubMed Identifier (PMID).

The process by which CTD selects articles for curation is a well-documented, two-pronged methodology. First, CTD biocurators review, cover-to-cover, 19 relevant toxicological journals on an ongoing basis [2]. Additionally, CTD selects other articles by targeting specific chemicals or chemical classes (e.g. bisphenol A, PFAS) from our Chemical Priority Matrix, and querying for them in PubMed [3]; returned articles mentioning the targeted chemical(s) are then ranked by CTD’s text mining pipeline, and the highest ranked are curated [4].

One of the core principles that underlies CTD curation is the use of highly structured, controlled terminologies throughout the database, not only for biomedical concepts (i.e. chemicals, genes, phenotypes, diseases, anatomical terms, species) but also for internally developed action terms that are used to form edge relationships between these biomedical concepts [3].

Utilizing this disciplined approach has allowed CTD to become highly computational, enabling it to not only be a knowledgebase, summarizing for scientists the state of research for one or more specific biomedical concepts in a systematic manner, but also a discoverybase, identifying prospective relationships between concepts that have not yet been reported. For example, if one study demonstrates that the chemical *Sildenafil Citrate* increases the expression of gene *ADIPOQ*, and another study shows that *ADIPOQ* is a potential target for the treatment of the disease *Myocardial Ischemia*, then we can computationally infer a hypothesis that *Sildenafil Citrate* could have a potential effect on *Myocardial Ischemia*. These types of prospective connections are available in CTD in the form of chemical-disease, gene-disease, and phenotype-disease inferences [5–7], and the former two are scored and ranked based on the topology of the local network used to generate the inference [8]. By extending this logic, we can assemble prospective four-unit stepwise molecular mechanisms, linking an initiating chemical, an interacting gene, an intermediate phenotype, and a disease outcome; these information blocks are known in CTD as *tetramers* [9].

This type of prospective computational chaining would not be possible in the absence of manual curation based on highly structured ontologies and controlled vocabularies (referred to hereafter as controlled vocabularies). At CTD, a subset of the “Chemicals and Drugs” [D] branch of the National Library of Medicine’s Medical Subject Headers (MeSH) [10] is used to characterize chemicals; a subset of National Center for Biotechnology Information’s (NCBI) Gene vocabulary [11] is used for genes; MEDIC [12], a hierarchical integration of the “Diseases” branch of MeSH and the Online Mendelian Inheritance in Man (OMIM) [13], is used for diseases; the Gene Ontology (GO; 14), particularly the biological process branch, has been successfully leveraged by CTD as a source to annotate chemical-induced phenotypes (defined at CTD as nondisease biological events) reported in the literature, such as vehicle emissions-induced increases in “leukocyte migration” (GO:0050900), cadmium-induced changes in “heart contraction” (GO:0060047), and arsenic trioxide-induced “gluconeogenesis” (GO:0006094) [7]; NCBI Taxonomy [15] is used for species; and the “Anatomy [A]” branch of MeSH is used for anatomical terms.

## Importance of term normalization at CTD

To the uninitiated, it might seem like a relatively simple proposition to curate within the context of a highly disciplined, structured environment; in reality, this is not the case. For example, the gene “aryl hydrocarbon receptor” may appear in the literature as *AHR, AhR 1, AHR1, AHR1A, Ahre, AH receptor, AH-receptor, aromatic hydrocarbon receptor, aryl-hydrocarbon receptor, arylhydrocarbon receptor, aryl hydrocarbon receptor 1*, etc.; it is the job of the biocurator to deduce, from raw text, the proper NCBI Gene primary term. The process biocurators use to translate raw text from the literature into controlled vocabularies is called *normalization* [16]. Structuring of this nature from free-form text tends to be an inherently tedious and time-consuming process, and curation efforts are typically resource-heavy [17].

The web-based CTD Curation Application (CAPP) is the ultimate arbiter of term normalization at CTD [3]. During the curation process our biocurators enter into the CAPP only primary terms, or unique synonyms to primary terms, within the context of the most recent version of the respective controlled vocabulary (which is typically less than a month old). The tool integrates a tightly controlled, internally developed notation to enable biocurators to quickly enter complex interactions. For example, the biocurator may want to note the following complex interaction based on review of a reference:

BAK1 protein affects the reaction [BAX protein affects the reaction [mirdametinib promotes the reaction [MK 2206 promotes the reaction [romidepsin results in increased apoptotic process]]]]

In order to capture this interaction, the biocurator will enter the following structured notation in the CAPP:

G1/p 1rxn [G2/p 1rxn [C1 +rxn [C2 +rxn [C3 +phe P1]]]]

**Figure 2.**
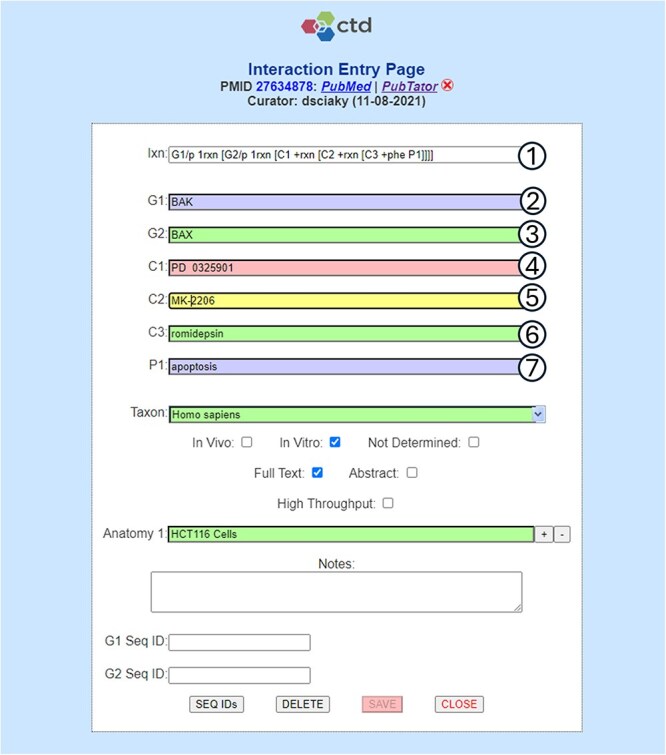
Term normalization during CTD curation application interaction entry. CTD biocurators use a structured notation as shorthand to capture an interaction (circle 1), in this case requiring the entry of two genes (G1 and G2), three chemicals (C1-C3), and one phenotype (P1). Once the notation is entered, labeled input fields (circles 2–7) are dynamically generated to allow the biocurators to enter the appropriate terms. In this example, the biocurator enters (circle 2) the gene *BAK*, which is the actual term used by the authors in the reference, for G1; however, *BAK* happens to be a nonunique synonym for the primary NCBI Gene term *BAK1* and, because of this, the entry is flagged as invalid (as indicated by the purple field background). The biocurator enters (circle 3) the correct primary NCBI Gene term *BAX* (with validation indicated by the green background). The biocurator enters (circle 4) chemical *PD 0325901*, which is an invalid entry (as indicated by the red background) because it contains two spaces between *PD* and *032*. The biocurator enters (circle 5) the valid chemical *MK-2206*, which is a unique synonym for primary MeSH term *MK 2206* (as indicated by a yellow field, which is used for valid unique synonyms). The biocurator enters (circle 6) the correct primary MeSH term *romidepsin*. The biocurator enters (circle 7) the nonunique, invalid synonym used in the reference, *apoptosis*, rather than the correct GO primary term, *apoptotic process*. The biocurator will be unable to save the interaction to the database until all the field entries are made valid (i.e. all the terms have been fully normalized to CTD’s controlled vocabularies, as indicated by either a green or yellow background).

This interaction involves two genes, G1 and G2, three chemicals, C1, C2, and C3, and one phenotype, P1. For each of these actors, the biocurator must translate the raw text from the literature into a primary term, or a unique synonym to a primary term (Fig. 2). The need for Natural Language Processing (NLP)-based tools to assist biocurators with this seemingly simple task of term identification, normalization, and entry (as well as other computational tasks) has been an important ongoing focus of our research and development at CTD.

## NLP research and development at CTD

Although CTD has been named a member of the “golden set of databases that have consistently served as authoritative, comprehensive, and convenient data resources” widely used by the scientific community [18], our staff has, since its inception, been comparatively small. In order to make the most of our limited resources, CTD’s team is relentlessly focused on improving the efficiency, effectiveness, and relevance of our curation [2–4]. Over a decade ago we recognized not only the aforementioned need for NLP-based tools to assist with the process of term identification and normalization, but also the fact that CTD could play an important role in the development of such tools based on our vast portfolio of manually curated content.

Consequently, CTD became an active member of the Critical Assessment of Information Extraction systems in Biology (BioCreAtIvE) group steering committee. BioCreative challenge evaluation tasks collectively represent an international biomedical community-wide effort, assembling NLP and biology expertise to develop and evaluate a variety of text mining and information-extraction systems within the biological domain [19]. Over the years, CTD team members have organized challenge evaluations and provided corpora for aggregate gene-, disease-, and chemical-based Named-Entity Recognition (NER) and document-level prioritization [20]; Web Services-based gene, disease, and chemical NER extraction [21]; and chemical-induced disease (CID) relation extraction [22]. CTD also worked closely with NCBI to assist with the development of BioC, a common, interoperable interchange format to represent, store, and exchange data computationally [23].

Concurrent with our community research-related efforts, we assembled and built our own NLP tools for internal use. Open source-related components were used at CTD to build a prototype document ranking application [24]. The prototype and associated tools were subsequently leveraged as the basis for development of a highly effective, fully functional, algorithmic rules-based text mining pipeline. This pipeline assigns document relevancy scores (DRS) to prospective articles, ranking each in terms of their suitability for curation within the context of specifically targeted chemicals, and has been demonstrated to increase productivity, efficiency, and the collection of novel content [4].

Integral to the ranking algorithm is NER extraction using a set of locally installed, third-party tools adapted for CTD use: Abner [25] for gene NER, Oscar3 [26] for chemical NER, and MetaMap [27] for disease NER, as well as supplementary chemical and gene recognition. Although the CTD text mining pipeline has been highly effective, we found that the problems inherent in local installation of NER tools are many, nontrivial, and multiply over time. As is typically the case with shared runtime libraries, local tool installation requires software engineers to address tool-specific issues such as operating system compatibility, third^-^party library requirements, process modularity and inter-process communications, programming language interpretation/compilation-related compatibility, and multi-thread capacity [21]. Moreover, libraries are static in nature; consequently, they are improved only with new releases, which, if implemented, may again give rise to many of the aforementioned technical issues.

This somewhat difficult experience with local libraries led us directly to our research associated with Web Service-based NLP. Web Services are services designed to accommodate interoperable machine-to-machine interaction over the Web [28]. Rather than locally installed libraries, Web Services provide the capability to make HTTP-based calls to remote sites for NER extraction. This approach tends to be inherently simpler than direct local integration because the technical details of the tools themselves are abstracted by the Web Service. The use of Web Services is particularly attractive for applications where asynchronous processing is a viable alternative, given that remote Web Service calls may or may not meet real-time curation application performance requirements.

Our Web Services-related BioCreative work demonstrated not only the dramatic advantages of remote tool integration, but also the utility of a standard, interoperable exchange format [21]. Use of an exchange format such as BioC is clearly superior to an output format that is tool-specific. In the latter case, unique format translation software must be written and maintained for every integrated library (as was the case with our text mining pipeline).

Finally, our NLP-related research and development experience underscored the importance of determining how well NER tools would comport to our controlled vocabularies. For example, our text mining pipeline library, MetaMap, maps disease, chemical, and gene terms to the National Library of Medicine’s Unified Medical Language System Metathesaurus [29]; Oscar 3 is tightly coupled with Chemical Entities of Biological Interest [30] for chemicals. Tools that map to non CTD-integrated controlled vocabularies, or tools that simply extract the actual terms used in the paper as biomedical concepts, require an additional layer of computational algorithms for term normalization, and are therefore less desirable than tools that map directly to CTD-integrated controlled vocabularies.

The experience and knowledge that we gained in conjunction with these projects enabled CTD to more judiciously pursue methods to integrate NLP tools into our curation process. These tools would be integrated in order to improve the efficiency and effectiveness of our curation staff, assisting our biocurators, as well as our computational processes, with identifying, normalizing, and entering chemical, gene, phenotype, disease, anatomical, and/or species (C-G-P-D-A-S) terms from the literature.

## Objectives for NLP tool evaluation

The general objectives in undertaking this project were to:

- Integrate into our curation process state-of-the-art NLP/NER tools for as many relevant C-G-P-D-A-S biomedical concepts as are available, using these tools as the basis for (i) creating a user interface for biocurators to view abstracts with clearly highlighted biomedical concepts, and (ii) ensuring the biomedical concepts are easily selectable, rather than requiring manually keying/copying-and-pasting for curation entry.
- Ensure the NLP tools align biomedical concepts as closely as possible with our underlying controlled vocabularies.
- Integrate NLP tools that: (i) are, if possible, available for use via a Web Service or similar web-based remotely accessible tool, rather than locally installed libraries, and (ii) employ an interoperable exchange format.
- Ensure that the tools are well maintained, production ready, and likely to be supported for the foreseeable future.

It should be noted that NLP tool-related response time was not an important factor in our evaluation. Of paramount importance was that whatever plan we implemented help speed curation, not slow it down. Being mindful of that principle, the assumption was that we would preprocess articles in an asynchronous fashion, storing the results in our database prior to curation, rather than running NLP tools synchronously during curation; it simply did not seem feasible to perform NLP processing for up to six different biomedical concepts (i.e. C-G-P-D-A-S) at run-time. Moreover, our plan was to integrate NLP tools into the CAPP as well as multiple text mining pipelines. Were we to perform all text mining processing synchronously, many of our references would be text-mined at least twice—one or more times during DRS scoring and later during actual curation. Therefore, it would be most efficient to store the results initially in our database during DRS scoring and ranking (for those articles requiring DRS scores); the NLP metadata extracted during DRS scoring would then be available for use by the CAPP during curation. Also, this preprocessing approach easily accommodates the potential integration of additional text mining tools into the curation process. For example, CTD is currently studying the use of Large Language Models for entity and relationship recognition; the metadata associated with these types of tools could be combined with PubTator metadata by storing the results in the database for later use without regard to the source. Although preprocessing the literature would be significantly more complex from a workflow and software engineering perspective, it was the approach that would ensure biocurators would not be adversely affected by NLP integration; consequently, preprocessing rendered the response times of the NLP tools of little significance.

## CTD/PubTator 3.0 compatibility

CTD had collaborated with members of the Literature Search group at NCBI on several of the aforementioned NLP-related projects and been highly impressed with their professionalism and expertise. We were aware that this group was responsible for PubTator Central [31], an online tool for viewing annotated concepts in biomedical literature, but had never had the opportunity, from a resource perspective, to carefully review PubTator in terms of its suitability for CTD. Given this knowledge and prior experience, we decided to evaluate PubTator for potential integration into CTD.

As we closely examined PubTator, we were pleased not only with its functionality and effectiveness, but also with its compatibility with CTD’s environment (Table 1). Similar to CTD, diseases and chemical names are normalized by PubTator to MeSH identifiers; genes are normalized to NCBI Gene identifiers; and species are normalized to NCBI Taxonomy identifiers [32]. Thus, four of the six C-G-P-D-A-S biomedical concepts we were interested in securing NLP support for were already addressed by PubTator directly, requiring no need for additional computational term normalization. Of note is that PubTator also uses NLP tools to identify cell lines and protein variants.

**Table 1.** CTD/PubTator controlled vocabulary overview. An overview of the key vocabularies used in CTD is provided, as well as how these vocabularies are addressed by PubTator. NOTE: In most cases, CTD uses a subset of the ontologies/controlled vocabularies

Biomedical concept	CTD-integrated ontology/controlled vocabulary	PubTator normalization	Direct PubTator/CTD normalization?
*Chemicals*	MeSH [10]	MeSH	✓
*Genes*	NCBI Gene [11]	NCBI Gene	✓
*Diseases*	MEDIC [12] (MeSH/OMIM [13])	MeSH	✓
*Phenotypes*	Gene Ontology [14]	Not applicable	Not applicable
*Species*	NCBI Taxonomy [15]	NCBI Taxonomy	✓
*Anatomical terms*	MeSH	Not applicable	Not applicable

The online user interface of PubTator is very thoughtfully designed. We had anticipated the need to develop an interface for our biocurators which highlighted C-G-P-D-A-S biomedical concepts based on the integrated NLP tools; fortunately, we discovered that this requirement was no longer necessary. Instead, PubTator, with its color-coded, selectable, and hyperlinked biomedical concepts, was more than adequate to address our near-term requirements. We had also expected that PubTator would provide solely abstract-based annotations but found that full text was provided where available. This full-text capability is extremely beneficial to CTD, because we are often required to review the full text in cases where the abstract is incomplete or requires further clarification. After evaluation and testing, we realized that it would be highly advantageous to integrate the PubTator online user interface component into our curation workflow; consequently, we modified the CAPP to hyperlink to PubTator (in addition to our existing PubMed hyperlink) and directed our biocurators to start using the PubTator online user interface as the basis for curation at their discretion.

We found that the strength of PubTator is not limited only to its online interface. In addition, the online tool is closely matched with a fully functional, Web Service-based Application Programming Interface (API); the API enables computational processes to extract, in a variety of methods and formats, the underlying metadata used to power/support the online tool. More specifically, PubTator’s API provides very detailed information about each processed reference and the associated biomedical concepts, including, for each concept, its spatial orientation in the paper, its type and accession identifier, and the raw text that was used to identify and normalize it.

In CTD, curated content is referenced via PMIDs. NCBI’s PubMed archive contains primarily abstracts from nearly all of the biomedical literature. Alternatively, PubMed Central is NCBIs full-text archive; it is indexed by PubMed Central identifiers (PMCIDs). PMCIDs are only available for a subset of PMIDs, primarily limited to those papers published in open access journals. A very useful extraction feature provided by the PubTator API is that applications need not have *a priori* knowledge of the PMCID to secure metadata associated with full text; knowledge of the PMID is sufficient for full-text extraction. Given that CTD is solely PMID-centric, the ability to extract full-text annotations using PMIDs is extremely beneficial.

The PubTator API supports three different output formats, including two that are BioC-based (BioC-XML or BioC-JSON). This was an important factor in our positive evaluation not only because CTD was already familiar with BioC, but also because we could address four of the six aforementioned biomedical concepts using a single format translating parser.

At PubTator’s core is AIONER, an effective, robust, cutting-edge deep learning NER transformer model, which is used to target and normalize the six aforementioned biomedical concepts supported by PubTator [33]. AIONER has been evaluated against 14 NER benchmark tasks; performance on these benchmark tasks validated that AIONER is an effective, robust tool, matching or surpassing previous state-of-the-art methods [31]. This was among our most important requirements, and we were very impressed anecdotally with how well AIONER performed.

In the final analysis, PubTator was not only met with overwhelming approval by the CTD curation team from a qualitative perspective, PubTator successfully fulfilled virtually every CTD evaluation target requirement (Table 2). The tool is state-of-the-art in terms of its effectiveness, addressing the full normalization of four of the six C-G-P-D-A-S biomedical concepts directly to its respective underlying CTD-integrated controlled vocabulary. It provides an online interface our biocurators can use for curation. The online tool is tightly coupled with a Web Service-based API that can be leveraged computationally to generate selectable drop-down lists of concepts for our staff. The interchange format for the APIs is BioC-based, greatly simplifying the translation to CTD. Finally, the tool is built and maintained by NCBI, a respected and, equally importantly, stable organization with which to partner for the long term. Given these factors, we determined that it was a better use of our limited resources to begin integrating PubTator into our curation workflow immediately rather than searching for and evaluating additional NER tools.

**Table 2.** CTD NLP objectives. The objectives associated with integrating state-of-the-art NLP/NER tools into the CTD curation process are summarized, along with a brief explanation of how each was satisfied by PubTator

CTD NLP integration project objectives	PubTator satisfaction of project objectives
Provide an online interface for biocurators to view abstracts with clearly highlighted biological concepts	Integral to PubTator is an online interface with color-coded, selectable, and hyperlinked biological concepts for both abstracts and full text (where available)
NLP tools align closely with CTD biological concept controlled vocabularies (see Table 1)	PubTator metadata maps directly to 4 of the 6 required biological concept vocabularies (see Table 1)
Biological concepts are easily selectable by biocurators	CTD CAPP was modified to include selectable PubTator metadata
Metadata is remotely accessible via a Web Service or similar web-based tool	PubTator metadata is accessible via a remote Web Service API; local tool installation is not required.
NLP tools employ an interoperable exchange format	PubTator provides multiple output formats, including BioC, an interoperable interchange format to exchange biomedical metadata computationally
NLP tools are well maintained, fully supported, and production ready	PubTator was developed and is supported by the National Center for Biotechnology Information, and is available via PubTator Central
NLP tools are state-of-the art	Integral to PubTator is AIONER, a robust, cutting-edge deep learning NER transformer model which has been proven to be highly effective when tested against 14 NER benchmark tasks

## CTD/PubTator 3.0 integration

**Figure 3.**
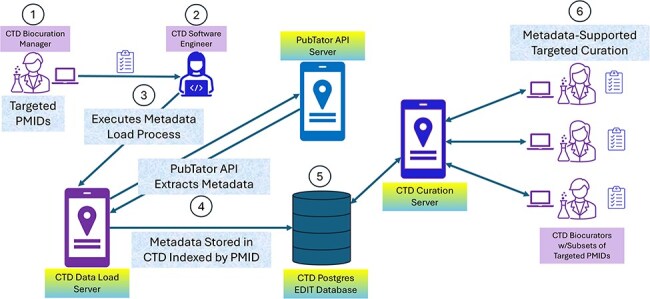
CTD/PubTator engineering architecture and workflow. In most cases, CTD identifies articles for curation by either targeting specific chemicals or chemical sets from a “Chemical Priority Matrix,” or curating articles from 19 important toxicological journals on an ongoing basis. CTD’s Biocuration Manager (circle 1) creates a list of PMIDs from these sources and forwards it to CTD software engineering for preprocessing. A member of the software engineering team (circle 2) takes the list and executes a process (circle 3) to extract metadata from PubTator by PMID using PubTator’s API; the metadata is returned to the process from PubTator in BioC-JSON format (circle 4). The metadata is then stored in a CTD Postgres database (circle 5). Once the preprocessing is complete, the Biocuration Manager assigns the PMIDs to the biocurators for curation. The CAPP computationally extracts the metadata from Postgres by PMID during curation (circle 6), assisting the biocurators with term identification and normalization.

The first step in the integration process involved implementation of a preprocessing paradigm (Fig. 3). As previously discussed, preprocessing provides the fastest online performance and thus is the most efficient option for our curation pipeline. We extract metadata in PubTator’s BioC-JSON format, forming the request using a concatenated string of PMIDs, and extracting full-text- or abstract-based biomedical concepts for genes, chemicals, diseases, and species. Each identified biomedical concept is reviewed to determine if it exists in CTD. Note that CTD does not store complete controlled vocabularies, but rather only those concepts that are relevant to our curation mission (e.g. our curation is limited to the *Eumetazoa* branch of NCBI Taxonomy, so only those species are included in CTD). The relevant metadata is then stored in two core PostgreSQL database tables, one organized by reference, and the other organized by biomedical concept within the context of reference.

**Figure 4.**
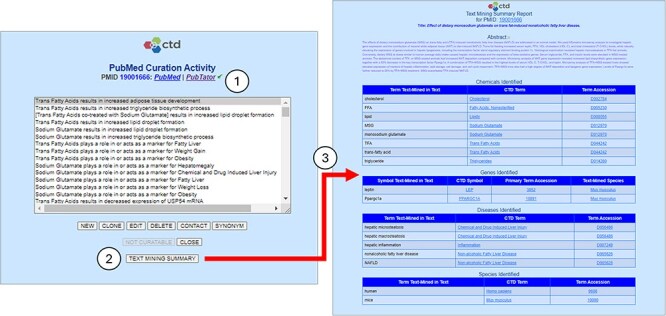
Text mining summary report. The CTD CAPP’s PubMed Curation Activity page provides links to both PubMed and PubTator; the green check mark (circle 1) indicates that the paper has undergone NLP-based preprocessing (conversely, a red “X” would indicate that the paper has not been preprocessed). The user may click on the *TEXT MINING SUMMARY* button to execute the Text Mining Summary Report for the respective reference (circle 2). The Text Mining Summary Report (circle 3) is organized into four biomedical concepts: chemicals, genes, diseases, and species. For each concept, the report provides not only the normalized terms (*CTD Term* columns) and associated accessions (*Term Accession* columns) identified by PubTator, but also the raw text upon which the term identification and normalization is based (*Term Text-Mined in Text* columns). For genes, the nominal species (per PubTator) is also provided (*Text-Mined Species* column).

The minimal modifications required to the CAPP’s user interface were implemented as nonintrusively as possible. A new “Text Mining Summary” button was added to enable users to execute a report which provides an overview of the NLP-related activity associated with the reference (Fig. 4). The report provides an option to view the abstract, either on the page or via a hyperlink to PubTator. It is segregated into four sections, one each for genes, chemicals, diseases, and species. The report provides only those terms integrated into CTD. Hyperlinks are provided for each biomedical concept to CTD and the respective source controlled vocabularies provider. PubTator not only identifies suspected genes that appear in the text, but also attempts to determine the species and appropriate accession of the gene (given that NCBI Gene accessions are species-specific); consequently, the report provides each gene’s primary term along with its nominal text-mined species.

Perhaps the most important feature of the summary report is that it provides the raw text from which PubTator identified and normalized each concept. This feature enables CTD biocurators to quickly determine which of the normalized concept translations they agree or disagree with based on the source text.

**Figure 5.**
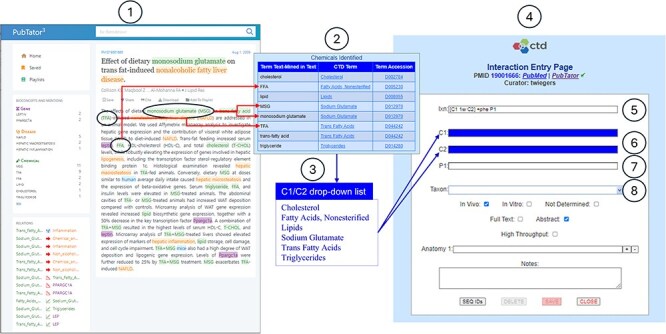
CTD/pubtator integration. CTD biocurators typically curate the literature using PubTator’s online interface (circle 1). An excerpt is provided of the Text Mining Summary Report (circle 2), which summarizes the chemicals identified by PubTator that are relevant to CTD. The fully normalized MeSH chemical terms are aggregated to create a distinct list of chemicals identified by PubTator (circle 3). CTD biocurators can use this list to enter an interaction during curation via the CTD CAPP’s *Interaction Entry Page* (circle 4). The biocurator enters structured notation into the *Interaction* field (circle 5) to indicate that two co-treated chemicals (C1 and C2) result in the increase of a phenotype (P1). The C1, C2, and P1 fields are dynamically generated to accommodate entry of the respective terms (circles 6–7). Note the deep blue background for the *C1* and *C2* fields (circle 6); this indicates to the biocurator that there is a drop-down list (circle 3) available for use; the biocurator may either pick from this drop-down list or manually enter any valid term. The *P1* field (circle 7) has a white background, indicating there is no associated drop-down list, so the biocurator must manually enter a valid term. The *Taxon* field (circle 8) always contains a drop-down list of the most curated species at CTD (i.e., *Homo sapiens, Mus musculus, Rattus norvegicus, Canis lupus familiaris, Danio rerio*, and *Oryctolagus cuniculus*); this list is supplemented, at the top, with any additional species identified by PubTator.

An overview is provided of how PubTator metadata is integrated into the curation process (Fig. 5). The process is largely unchanged with one major exception: Rather than biocurators having to normalize and then enter terms into chemical, disease, gene, and species fields, drop-down lists are provided containing the fully normalized, CTD-validated concepts provided by PubTator. Biocurators may choose to select entries from those drop-down lists or enter their own valid terms. Similar auto-fill, drop-down schemes have been found to have accelerated task completion time by 49% compared with manual normalization and entry [17].

## Results

Table 3 provides an overview of some key metrics associated with PubTator integration based on a review of the first 661 references curated by CTD that were preprocessed using PubTator. Of the 661 references surveyed, PubTator identified 8960 biomedical concepts, of which 4329, or 48%, were actually used by CTD biocurators for interaction curation. The breakdown by biomedical concepts is also provided. Of note, 69% of genes identified by PubTator were actually used for curation. This is important because identifying the correct gene symbol is the most time-consuming aspect of CTD biocuration. Review of full text is normally required to resolve the official gene symbol and species information; synonyms and alternative names, reactive monoclonal antibodies, DNA sequences, derived RT-PCR primers, accession identifiers, and/or citations mentioned by the authors are typically used to identify official gene symbols [34]. Consequently, the effective normalization of genes tends to significantly increase curation efficiency.

**Table 3.** CTD/PubTator integration statistics. Key metrics associated with the first 661 references that were curated by CTD using PubTator are provided. More specifically, counts for the biomedical concepts identified by PubTator, counts for the concepts that were actually used by CTD during curation, and the percentages used, are provided for each biomedical concept category. As well, the average size of the drop-down lists in CTD’s CAPP containing PubTator-identified biomedical concepts are provided.

Biomedical concept	Articles containing concept	Concepts identified by PubTator	Concepts identified by PubTator curated	% concepts curated	Avg drop-down list size
*Chemicals*	652	2893	1465	51%	4.4
*Diseases*	575	2220	302	14%	3.9
*Genes*	588	2989	2068	69%	5.1
*Species*	443	858	494	58%	1.9
*Totals/Avgs*	661	8960	4329	48%	3.8

Although CTD curated approximately 48% of identified biomedical concepts, this does not imply that the remaining 52% are incorrectly identified by PubTator. On the contrary, CTD biocurators have found PubTator to be highly effective at identifying key biomedical concepts, confirming anecdotally the results of the aforementioned PubTator benchmark testing. There are, however, many instances where PubTator correctly identifies a biomedical concept that does not fall within the necessary requirements for CTD curation. For example, an abstract may include the following sentence: “Noncoding RNAs including long noncoding RNAs (lncRNAs) and microRNAs (miRNAs) have been documented to play prominent role in neurodegenerative diseases including Parkinson’s disease (PD).” In this case, although PubTator may correctly identify “neurodegenerative diseases” and “Parkinson’s disease” as cited diseases, the sentence contains no association between the cited diseases and specific chemicals or genes; consequently, it will not be used for CTD curation.

## Limitations and future directions

We have not yet completed all of the desired objectives with respect to this project; we still expect to integrate NER tools into our curation process for anatomical terms and phenotypes (i.e. GO). Although tools exist for these entity types [35, 36], we need to conduct further research before defining the best solution. Once potential tools are identified, they will be analyzed against CTD’s aforementioned NLP-related objectives; tools which most closely match our criteria will then be integrated into applicable pipelines. In preparation for addressing these requirements, we have built our software engineering architecture around the need for the integration of additional NER tools; consequently, the preprocessing paradigm and database schema design lends itself nicely to the future integration of additional tools.

We also plan to develop two new text mining pipelines. First, we plan to re-write our existing pipeline, which addresses core curation only (chemical-gene, chemical-disease, chemical-phenotype, gene-disease, gene-phenotype), replacing it with a new one which integrates PubTator metadata. Second, we plan to write a new, radically different pipeline which leverages the PubTator metadata specifically for the ranking of prospective articles related to our Exposure module [37].

Finally, we plan to determine whether it would be beneficial to integrate specific entity relationship-based metadata provided by PubTator into CTD’s CAPP. Currently, it is an open question whether these relationships would be directly applicable to CTD curation.

## Summary

PubTator is exceptionally well designed, from both an end-user and technical perspective. The features provided were comparatively straightforward to integrate into CTD’s well-defined curation workflow. It is enormously beneficial to small groups like CTD that NCBI has had the foresight to develop tools that can be relatively easily leveraged to secure state-of-the-art performance. In the absence of tools like these, CTD would simply lack the resources necessary to fully harness the power and efficiency of NLP. Groups that could benefit from the features provided by PubTator would be well-advised to evaluate it against their specific requirements.

## Technical environment

CTD primarily utilizes a Jakarta EE-based, Model-View-Controller architecture, integrating POJOs/JSPs/JavaScript/servlets in conjunction with PostgreSQL database management systems, all within the context of Linux and Apache/Tomcat application servers.

## Data Availability

The data underlying this article will be shared on reasonable request to the corresponding author.
